# The processing of intimately familiar and unfamiliar voices: Specific neural responses of speaker recognition and identification

**DOI:** 10.1371/journal.pone.0250214

**Published:** 2021-04-16

**Authors:** Julien Plante-Hébert, Victor J. Boucher, Boutheina Jemel

**Affiliations:** 1 Laboratoire de Sciences Phonétiques, Département de Linguistique et de Traduction, Université de Montréal, Montréal, QC, Canada; 2 Laboratoire de Recherche en Neurosciences et Électrophysiologie Cognitive, Hôpital Rivière-des-Prairies, Montréal, QC, Canada; 3 École d’Orthophonie et d’Audiologie, Faculté de Médecine, Université de Montréal, Montréal, QC, Canada; University of California, Los Angeles, UNITED STATES

## Abstract

Research has repeatedly shown that familiar and unfamiliar voices elicit different neural responses. But it has also been suggested that different neural correlates associate with the feeling of having heard a voice and knowing who the voice represents. The terminology used to designate these varying responses remains vague, creating a degree of confusion in the literature. Additionally, terms serving to designate tasks of voice discrimination, voice recognition, and speaker identification are often inconsistent creating further ambiguities. The present study used event-related potentials (ERPs) to clarify the difference between responses to 1) unknown voices, 2) trained-to-familiar voices as speech stimuli are repeatedly presented, and 3) intimately familiar voices. In an experiment, 13 participants listened to repeated utterances recorded from 12 speakers. Only one of the 12 voices was intimately familiar to a participant, whereas the remaining 11 voices were unfamiliar. The frequency of presentation of these 11 unfamiliar voices varied with only one being frequently presented (the trained-to-familiar voice). ERP analyses revealed different responses for intimately familiar and unfamiliar voices in two distinct time windows (P2 between 200–250 ms and a late positive component, LPC, between 450–850 ms post-onset) with late responses occurring only for intimately familiar voices. The LPC present sustained shifts, and short-time ERP components appear to reflect an early recognition stage. The trained voice equally elicited distinct responses, compared to rarely heard voices, but these occurred in a third time window (N250 between 300–350 ms post-onset). Overall, the timing of responses suggests that the processing of intimately familiar voices operates in two distinct steps of voice recognition, marked by a P2 on right centro-frontal sites, and speaker identification marked by an LPC component. The recognition of frequently heard voices entails an independent recognition process marked by a differential N250. Based on the present results and previous observations, it is proposed that there is a need to distinguish between processes of voice “recognition” and “identification”. The present study also specifies test conditions serving to reveal this distinction in neural responses, one of which bears on the length of speech stimuli given the late responses associated with voice identification.

## Introduction

The ability to recognize and identify voices stands as a remarkable and yet puzzling process of speech perception. From an evolutionary perspective, this ability is said to have been vital for the survival of humans and other species [1]. But when one recognizes a voice, it is usually in the context of speech. No other species processes voice information in the context of fluctuating sounds of oral articulations, and the human ability to recognize or identify voices in such a context can be quite robust. In fact, in the case of an intimately familiar voice, such as the voice of a parent or sibling, there is nearly perfect recognition or identification independently of visual information [2]. It is frequently assumed in voice research that such accuracy rests on the sensory processing of the spectral attributes of a voice signal as when producing such sounds as “ahhh” where oral motions are minimized [e.g., 3, 4]. However, as we established in an earlier study, when listeners are asked to pick out an intimately familiar voice amongst unfamiliar or unknown voices with similar fundamental frequency (F0), there is a degree of inaccuracy when the stimuli are single syllables [5]. For near perfect recognition and identification to occur, two or more syllables can be required, and nasal sounds can be a factor for short sequences [6–13]. This suggests that the processing of speaker-specific voice information involves dynamic spectro-temporal attributes reflecting moving resonators rather than static voice harmonics. It also indicates that, while some processing of speaker-specific information rapidly occurs over short intervals of speech, correct recognition or identification can require slightly longer temporal spans. Of course, given such findings, any attempt to circumscribe differing neural correlates of voice processing requires techniques that offer high temporal resolution (such as electroencephalography, EEG, and magnetoencephalography, MEG). It also entails, for the sake of clarity, a terminological distinction between processes that can potentially apply over different time intervals.

Indeed, the lack of a formal distinction between processes, or the variable use of terms such as voice “discrimination”, “recognition”, and “identification” to refer to an undefined “speaker identity” has created a degree of confusion in the literature. The terms have been used to designate fundamentally different processes and can thus be essential in understanding the neurological mechanisms that underlie the processing speaker-specific voice information. As repeatedly pointed out by Hanley et al. [14–16], a terminological distinction between these processes is especially crucial in voice research since episodes of recognition in the absence of identification are much more frequent in the perception of voices than faces. The following section serves to outline previous findings and general principles that support a strict distinction between voice *recognition* and *identification*, and also provides a demonstration of how this distinction relates to different EEG components in response to intimately familiar and trained (to-familiar) voices. For the sake of clarity, we use separate terms to designate these and other types of vocal stimuli, including intimately familiar voices (IFV), familiar voices (FV), frequently presented or trained-to-familiar voices (TV), and unfamiliar or unknown voices (UV).

### On the neural underpinnings of voice discrimination, recognition, and identification

#### Early findings and clinical observations

Clinical reports in the 1980’s provided crucial insights that have guided research on the processing of speaker-specific voices. The condition associated with an impaired ability to recognize FVs first appeared under the name of “phonagnosia” in Van Lancker and Canter [17], a designation still widely used today. Since then, phonagnosia cases have been classified in two major categories: apperceptive phonagnosia, where the deficit is seen at the sensory or perceptual stages of voice processing, and associative phonagnosia, where the deficit lies in the association between a perceived voice and a particular speaker [18–20]. It is useful to note with respect to clinical reports that, until quite recently, all cases of phonagnosia were observed on patients with brain damages. However, Garrido, Eisner [21] presented a case study of developmental phonagnosia.

Early reports also focused on the ability of listeners to distinguish between FVs and UVs although “familiar” voices in these reports often referred to voices of famous individuals. The processing of these types of voices was generally investigated using tasks involving two-alternatives forced-choice paradigms [2AFC; e.g., 22]. Moreover, investigations of the processing of UVs typically used designs where dyads of voices were presented in tasks requiring discriminatory same/different or old/new judgments following in-lab learning [e.g., 23]. Using such protocols with participants presenting various brain lesions, Van Lancker, Kreiman [24] and Van Lancker and Kreiman [25] established that FV recognition can occur even when participants present an impaired ability to discriminate between pairs of UVs. This led the authors to conclude that sensory discrimination of unfamiliar voices could not be a preliminary stage of familiar voice recognition. Instead, the two abilities reflected different neural processes that were applied in parallel and not in a particular sequential order [25].

Following this line of research, later studies suggested that the processing of UVs rests on the perceptual processing of specific acoustic indices of pitch, speech rate, voice quality (etc.). According to these studies, the processing of voices involves a comparison of acoustic indices to prototypes stored in long-term memory and which come to consolidate in memory through a repeated exposure to voices [see 26–28]. In this view, then, the discrimination of FVs and UVs centers on a presumed process of comparison between heard acoustic features of different voices and particular features coded in long-term store.

When it comes to familiar voices, however, an important distinction needs to be made between the feeling of knowing a stimulus and being able to explicitly recall qualitative information about the stimulus. In psychology, this principled difference is captured by general terms of “familiarity” and “recollection” [29–31]. Such a distinction is generally admitted in memory research and supported by neurophysiological observations [32–34]. Recollection, or recalling information about a stimulus as compared to judgments of its familiarity, involves episodic memory which is generally seen to entail activity in frontal cortical regions [35, 36]. In this light, reviews of the clinical literature on the processing of FVs and UVs have indicated that distinct neural mechanisms underlie the feeling of familiarity as compared to the retrieval of episodes that have consolidated to form semantic representations relating to voice or speaker identity [37, 38]. Thus, the feeling of having heard a voice and knowledge of who a speaker is entail different processes, which implies that investigations of these processes require different types of voice stimuli. Within early and current voice research, the terms voice *recognition* (familiarity) and *identification* (involving the retrieval of semantic information) should be regarded as a principled distinction by which to understand voice processing, as suggested by Kreiman and Sidtis [39]. But this entails that stimuli consisting of previously heard or marginally familiar voices (FVs), including trained-to-familiar voices (TVs), may not necessarily involve identification processes as in *intimately* familiar voices (IFVs).

In their model of voice identity processing, Kreiman and Sidtis [39] propose that UVs are processed in terms of characteristic features while FVs are processed as whole “Gestalt-like” patterns. Hemispheric specialization, as described in Kreiman and Sidtis [39], varies specifically with voice familiarity. The view is that comparisons of features, which occurs in discriminating and recognizing UVs, links to processes in the left hemisphere whereas pattern-like recognition and identification of FVs involves functions of the right hemisphere. This distinction between FVs and UVs in terms of pattern and feature processing has also been supported by a number of recent studies reviewed by Stevenage [40]. In sum, the aforementioned differences between voice discrimination, recognition, and identification, as well as between types of voice stimuli (IFV, FV, TV, and UV) appear essential to circumscribing different neural mechanisms involve in processing vocal attributes of speech. Yet such distinctions, especially between voice recognition and identification, are not generally reflected in voice research. This can lead to a degree of confusion in interpreting observations in terms of underlying neural processes over and above differences in methodology, as illustrated below.

#### Electrophysiological observations

In considering studies that use ERPs, the following brief review sets aside a body of work relating to the interplay of visual and vocal information in voice processing, which entails varying methodologies [for summaries of this work, see 41–44]. Early studies involving ERPs focused on the discrimination of human voices and non-human sounds (e.g., animal cries, bell sounds, tones, etc.), which showed distinct responses to voices with an onset as late as 400 ms or the N400 [45–47]. More recent reports have revealed that the discrimination of human voices compared to generic sounds is represented by early components, around 150 ms, which have come to be termed the “fronto-temporal positivity to voice” [FTPV; 48–51]. Thus, there is evidence that the earliest neural components that relate specifically to human voices are in the order of 150 ms post-onset. Given these results, one can logically assume that any processing of voice identity information would entail later-occurring ERPs as could be revealed on stimuli of IFVs.

Few studies, however, have investigated the processing of IFVs such as the voice of a close friend or family member. One exception is a study by Beauchemin, De Beaumont [3]. That ERP study focused on responses of listeners to IFVs (close relatives or long-time friends) using an auditory oddball paradigm in reference to the MMN components [52]. Short speech samples consisting of single vowels lasting some 200 ms produced by familiar and unfamiliar speakers (IFVs and UVs respectively) were presented in conditions of passive listening. The results showed distinct responses across IFVs and UVs peaking at 200 ms post-onset, in the MMN range. Similar results of MMNs have also been reported in studies involving newborns, suggesting that the ability to recognize voices arises in early development [53–55].

In a different study that also involved an auditory oddball paradigm, Graux, Gomot [56] compared the ERP of three sets of presented voices, including FVs, UVs, and participants’ own voices (designated as “self”). The results displayed a significant MMN between 180 and 210 ms post-onset (for FVs compared to UVs) and a significant difference on the P3a between 230 and 320 ms for FVs compared to self-voice. These results confirmed a previous report of a distinct process between self and familiar voices [4]. On the other hand, given that externally generated familiar voices are never heard as self-generated voices, it is difficult to extrapolate results on self voices to processes of voice recognition and identification [and see 57–60]. On their side, Holeckova, Fischer [61], after exposing participants to their own name pronounced by intimately familiar and unfamiliar speakers, reported a small effect on the P3, between 300 and 380 ms, but mostly on later-occuring ERP between 625 and 800.

Other studies have investigated ERPs to IFVs but with quite different results bearing specifically on voice identification. Of particular interest is a study by Schweinberger, Walther [62] based on a 2AFC task involving paired stimuli of two IFVs that were morphed to varying degrees with one another. ERPs in this paradigm reflected changes in voice identification when increasing the proportion of one IFV in the stimuli relative to another IFV. The experiment also included congruent/incongruent speech contexts with /aba/ and /iɡi/ serving to examine the effects of verbal contexts on responses. Importantly, the results showed two responses occurring at different time intervals. A first response to IFVs occurred at parietal sites starting at 250 ms post-onset when speech contexts were congruent whereas, when speech contexts were incongruent, a speech-independent response to IFVs appearing, not as short-time components, but as protracted changes between 300 ms and 600 ms post-onset, in the P3 range. It is useful to note that the authors used the designation “voice identification” in commenting on their results (while also expressing reservations on their interpretations owing to the small number of participants).

Another investigation that involved ERPs and more or less FVs was that of Gonzalez, Bobes Leon [63]. Their experiment used a go/no-go task with presented FVs and UVs in the context of a short phrase (the Spanish word /ola/). In this case, “FVs” referred to participants’ friends or colleagues so that it is unclear whether the stimuli could qualify as IFVs. The results showed ERP differences between FVs and UVs appearing between 280 and 840 ms post-onset, including a N250r and a P3, but again reflected protracted responses rather than short-time components as in Schweinberger, Walther [62].

Finally, one should note that it is often assumed in voice research that pitch (given by F_0_) is voice-specific whereas such aspects can relate to speech processing as in the case of “tone languages” where pitch changes serve to distinguish between words. In a study involving ERPs (and fMRI), Zhang, Pugh [64] examined the varying responses obtained when listeners attend to changing lexical tones in two Cantonese words /ji/(produced with high or rising tones) and when they attend to changing voices (UVs of a male and female speaker producing the words). The design aimed to compare ERPs of talker and speech deviants with reference to a standard. The analyses of designated components showed talker-specific changes in P2, P3a, and on frontal negativities examined over an interval of 500–800 ms (parietal late components also appeared but were not analyzed). An important methodological implication of this study is that it showed task-dependent interactions between talker and speech processing where pitch could not be taken *a priori* as a property of “voices”. Moreover, the authors specified that the differences in F_0_s between the male and female voices (101 Hz) exceeded differences in F_0_s of speech contexts (56 Hz). There is much behavioral evidence that salient differences in voices can influence memory as opposed to less distinctive voices and such differences on distinctiveness are likely to reflect in ERPs. However, few reports specify F_0_ values of voice stimuli, which may underlie the discrepancy in reported components of voice processing. But perhaps a more important source of variation is the length of the stimuli used across studies.

Generally, and in comparing various reports listed in Table 1, ERP responses to IFVs appear to involve short-time components between 150 and 320 ms but also prolonged responses with latencies up to 840 ms that have not been identified in terms of specific components. Although several methodological factors may underlie the discrepancies in reported latencies, one basic factor appears to be the duration of the stimuli, as seen in Table 1. In terms of reports involving IFVs, the stimuli length in studies by Schweinberger, Walther [62] and Gonzalez, Bobes Leon [63] provided sufficient dynamic spectral information so as permit speaker identification, whereas it can be questioned whether single vowels offer sufficient sensory information for this process [see 6].

**Table 1:** Summary of ERP studies of voice processing arranged by type of stimuli and types of voices–intimately familiar voices (IFV), famous/familiar voices (FV), trained-to-familiar voices (TV) or unfamiliar/unknown voices (UV). Only time windows in relation to voice processing are reported in the table.

Reference	Voices	Stimuli	Component(s)	Latency (ms)
Beauchemin, De Beaumont [3]	IFV / UV	/a/	MMN, P3	200, 240–320
Graux, Gomot [56]	IFV / UV	/a/	MMN, P3a	180–210, 230–320
Gonzalez, Bobes Leon [63]	IFV / UV	/ola/	N250r, P3	280–840
Holeckova, Fischer [61]	IFV / UV	530 ms name	P3, Slow waves	
Schweinberger, Walther [62]	IFV / IFV	/aba/ /igi/	P3	250–600
Schweinberger [65]	FV / UV	2000 ms speech	Sustained potentials	450–800
Schall et al. (2014)	TV	2-syll. words	N/A	200
Humble, Schweinberger [66]	TV / UV	1719 ms speech	Old/new effet	500–800
Zäske et al. (2014)	TV / UV	8-syll. words	N/A	290–370
Föcker, Hölig [67]	UV / UV	2-syll. words	N/A	270–530
Föcker, Best [68]	UV / UV	2-syll. words	N/A	200–250
Spreckelmeyer (2009)	UV / UV	Sung tones	N/A	300–400

As for investigations that focus on stimuli classed as TVs and “famous” FVs, these stimuli involve, respectively, UVs that become familiar during a training phase of an experiment, or FVs from celebrities. Importantly, an experiment by Schweinberger [65] using a priming paradigm established that priming voices before the presentation of FVs or UVs creates a response at 200 ms post-onset indicating a voice-recognition response. However, a speaker-identity response for famous FVs was only observed in a time window between 450–800 ms (although the author did not label these sustained potentials identification responses). Contrasting with these results, several reports using TVs have not revealed responses in windows beyond 450 ms. Thus, the MEG study of Schall, Kiebel [69], based on TVs, used long sentence-length stimuli. After learning six voices with corresponding names, participants were asked to indicate if a speech sample and a name were matching or not. Significant responses to speaker identity were observed at 200 ms post-onset. Zäske, Volberg [70] similarly reported a significant difference in ERPs using an old/new task with TVs and long stimuli. TVs that were correctly identified elicited a greater positivity than UVs starting at 300 ms post-onset, although how this reflected a speaker-identity response was unclear since the responses occurred on identical linguistic stimuli (i.e., it was unclear whether identity information was processed independently of verbal contexts). A following study reported in Humble, Schweinberger [66] reported a similar old/new effect bearing on speaker identity, but this effect was observed later (500–800 ms) and was elicited following the presentation of stimuli different at learning and at test. Spreckelmeyer, Kutas [71] also reported a voice recognition response at around 300 ms post-onset during a same/different task involving pairs of UVs. Consistent with these results, Föcker, Hölig [67] reported rising negativity starting at 270 ms post-onset for person-incongruent dyads of TVs compared to person-congruent ones. Yet, in a very similar study, Föcker, Best [68] found a significant response to paired TVs in time windows between 200 to 250 ms and 350 to 550 ms. However, with the exception of Schweinberger [65], it is unclear how the paradigms in the preceding reports serve to distinguish responses bearing on a processing of speaker-identity information from those that reflect a *recognition* of voices. In fact, in many of the reports the terms voice recognition and voice or speaker identification are used interchangeably or with vague definitions.

Overall, neural responses that relate to the recognition of TVs appear to occur in the range between 200 and 370 ms post-onset (see Table 1). The experiments of Schweinberger [65], using FVs, yielded much later responses that could be related to speaker identification. This also applies to the report by Gonzalez, Bobes Leon [63]. In comparing these studies to others in Table 1, one notices that the reported long latencies ranging from about 500 to 840 ms post-onset appear for speech contexts consisting of at least a few syllables. As emphasized by many authors, stimuli length affects speaker recognition and identification in that longer stimuli generally entail greater phonetic variability and spectro-temporal information [6, 7, 12, 13] (see also [72] and [73] for further discussion on this topic). In understanding the differences between responses at long latencies and those that occur at about 200–370 ms, it should also be weighed that stimuli of famous FVs can associate to varying degrees with a multimodal episodic memory of speakers, whereas TVs, which are experienced in a laboratory setting or through repeated audio presentations, may not serve to constitute such multimodal representations. This is not an issue when using IFVs where sensory experiences spanning years associates with the voice of an individual. Such differences could well underlie the separate responses across 200–370 ms and 500–840 ms where the first response reflects voice recognition and a later-occurring response may reflect a processing of identity information that bears on episodic or semantic memory of a speaker. However, it remains unclear whether this is actually the case given that, except for Schweinberger [65], studies have not compared responses to TVs and IFVs. In interpreting the time windows reported in Table 1, it is interesting to note that Schweinberger, Walther [62] is the only study where voice identification was associated with ERP response between 250 and 600 ms post-onset. As noted, the two other studies where voice identification possibly occurred, Schweinberger [65] and Gonzalez, Bobes Leon [63], showed responses ranging from about 500 ms to 840 ms. One potential explanation for earlier response times reported by Schweinberger, Walther [62] is that all presented voices were IFVs although participants did not specify if they knew the speaker and were aware that any of the voices they would hear was and IFV. This accurate prediction could have facilitated the identification process and therefore fasten the EEG response.

### The present study

In terms of the above research, one can surmise that EEG/MEG investigations of voice processing have not circumscribed the time course of fundamentally different processes of voice recognition and voice identification. Moreover, as summarized in Table 1, few studies focus on IFVs using sufficiently long speech samples that support accurate speaker identification [6]. Of the studies that do use stimuli consisting of at least a few syllables, separate responses appear on different time windows. Thus, while IFVs elicit responses in a 150–320 ms window, they also associate with prolonged responses as late as 840 ms post voice onset. The above discussion suggests that one reason for these prolonged responses is that IFVs and FVs carry information that links to semantic memory of a speaker such that the late responses reflect a process of voice identification.

The present study aims to bring further evidence supporting this latter view by examining the following prediction. Specifically, it is hypothesized that IFVs, compared to TVs and UVs, elicit voice recognition responses in a window of 150–320 ms, in the range of the P2 ERP component, as well as later-occurring responses extending beyond 450 ms, encompassing slow ERP waves, suggesting a distinct process of identification. This prediction also serves to clarify the effects of different types of voice stimuli, which are often indiscriminately associated with recognition and/or identification. Studies have frequently suggested similar responses for known voices regardless of whether these are IFVs, FVs, or TVs (as outlined in Table 1). Yet, as Kreiman and Sidtis [39] note, IFVs are distinctly processed, which should reflect in differential neural responses. It should be noted, however, that reports confirming these differential responses point to changes over long time frames (roughly 500–840 ms) and not to particular short-time ERP component [as in 63]. Indeed, studies of responses to IFVs that refer to components such as MMNs and FTPVs have used brief stimuli like single syllables which, as noted, may not provide sufficient information for processing voice identity [cf. 3]. For this reason, the present research is not driven by an assumption of particular components but instead explores how IFVs, TVs, and UVs elicit differential electric brain responses reflecting distinct processes of voice recognition and identification.

## Method

### Participants

Thirteen participants (8 females), aged between 21 and 43 years (mean = 30.81, s.d. = 5.14) completed the study. They were all native speakers of Quebec French except one speaker who learned Quebec French at four years of age. All were dominant right handers according to a standard questionnaire [74] and had normal hearing as established by an audiometric screening test. A forward and backward digit-span test [WAIS-III, 75] confirmed normal memory performance for all participants. It should be noted that participants recruited in the present study were selected following the recommendation of a member of an original pool of 36 male volunteers from whom voice samples were recorded and analyzed to create the stimuli in the present study [2]. Each volunteer in this pool provided the names of a family members, close friends, or life partners. The recruitment of participants in the present study was limited to these named individuals who could be matched to one target IFV in a set of otherwise unfamiliar voices but where all voices reflected speakers with similar Speaking Fundamental Frequency (SF0) to within one semitone, as described subsequently. The “intimate familiarity” of a target IFV was established via a questionnaire and criteria that were elaborated in a previous behavioural study [2]. The fact that participants were selected by reference to an IFV which had to be similar to other unfamiliar voices in a set restricted the recruitment to a small number of specific individuals (i.e., participants that could be matched to one IFV in a set of highly similar voices). All participants were paid, and written informed consent was obtained following guidelines of the Ethics Committee of *CIUSS du Nord-de-l’île-de-Montréal* at *Rivière-des-Prairies Hospital* (Montreal, QC) which also approved the present research.

### Stimuli

The voice stimuli were eight four-syllable utterances reflecting usual speech and expressions. These utterances, listed in Table 2, were produced by 12 native speakers of Quebec French who, as noted above, all had similar SF0 and had no discernible regional accents. The length of the stimuli (4 syllables) was decided following the results of Plante-Hébert and Boucher [6] and other observations relating to the length of contexts required for accurate speaker identification [7, 12, 13, 22, 72, 76]. These studies, especially the one from Plante-Hébert and Boucher, refer to stimuli exceeding one syllable for correct identification. Average SF0 was controlled and similar across the voice stimuli used in the experiment (the stimuli were spoken with a neutral intonation and cross-speaker differences in SFO for the voice samples did not exceed one semitone).

**Table 2:** The four-syllable utterances used as voice stimuli. Transcripts in regular orthographic Quebec French and IPA.

Utterance stimuli	IPA transcription	Nasal segments
*Bonjour madame*. *(Good morning mam)*	[bɔ̃ʒuʁmadam]	3
*Combien t’en prends*? *(How much do you want*?*)*	[kɔ̃bjɛ̃tãpʁã]	4
*Comment qu’elle va*? *(How is she*?*)*	[kɔmãkavɑ]	2
*De temps en temps*. *(From time to time)*	[dətãzãtã]	3
*Donne-moi en deux*. *(Give me two of them)*	[dɔmwazãdø]	2
*J’en connais quatre*. *(I know four of them)*	[ʒãkɔnɛkat]	2
*Quand est-ce qu’il vient*? *(When is he coming*?*)*	[kãtɛskivjɛ̃]	2
*Quelqu’un t’attend*. *(Someone is waiting for you)*	[kɛkœ̃tatã]	2

Finally, as indicated in Table 2, each utterance contained a number of nasal sounds, which have been shown to facilitate speaker identification, likely because they provide additional information on speaker physiology in relation to resonance cavities [5, 8–11, 77]. The voice stimuli were produced in a conversational fashion at steady rates and recorded in a sound-treated booth using an omnidirectional headset microphone (*C477 WRL*, AKG) and a 16-bit external sound card set to a sampling rate of 44,1 kHz (*Fast-track Ultra*, M-Audio). While recording these stimuli, the speakers produced each utterance after listening to an audio pacer consisting of separate tones. This ensured the production of similar timing and prosody across utterances. The recorded signals were amplitude normalized and each stimulus was segmented as a separate audio file. The onsets of the speech signals in the audio files were aligned so that the perceptual-center (*P-center*) of the first syllable of all utterances was at 200 ms from the beginning of the file. Alignment in terms of *P-centers* [described in 78, 79] insures that perceptual onsets of speech stimuli are stable and reduces jitter in EEG responses at the onset [see 80]. The overall length of the signals ranged from 618 ms to 1085 ms (mean of 818 ms, SD of 83 ms). Overall, the stimuli used in the present experiment respected generally admitted guidelines for the elaboration of voice line-ups in forensic applications [81–84].

### Pre-test stimuli validation

As a preliminary verification of the stimuli used in the present study, we conducted a pretest involving four volunteers that did not know any of the presented voices. The purpose was to establish whether equal numbers of presentations of the different voices and utterance contexts created non-specific ERPs. The test conditions were the same as during the experiment described below and each volunteer was exposed to a total of 10 trials per voice per utterance. The pretest confirmed that, in presenting different voices an identical number of times, average ERPs did not visually differ across utterance contexts. However, one of the voices had to be removed due to an unexplained difference in ERPs compared to the other voices. The pretest also confirmed that the multiple presentations of the different utterances did not have an effect on average ERPs across voices. In sum, variations in ERPs under the present test conditions can be related specifically to familiarity and frequency of presentation rather than utterance contexts or vocal idiosyncrasies.

### Procedure

Audio files containing the stimuli were arranged in eight blocks, each reflecting a specific utterance of Table 2. Within each block, the voices were randomized with the restriction that no consecutive presentation contained the same voice. Of the eight blocks of stimuli, four served to record EEG responses, and these alternated with four blocks that served to collect behavioural responses on speaker identity. Specifically, the EEG-recording blocks were ordered such that the first, third, fifth, and seventh blocks each contained 240 trials of passive listening. The four other alternating blocks each contained 60 trials where listeners identified the IFV using a key press. The latter blocks of trials were reduced in number so as to limit the overall test duration while allowing to collect behavioral confirmation of IFV identification. All blocks bore presentations of different types of voices in varying proportions: a frequently presented IFV (33.33% of trials), a frequently presented TV (33.33% of trials), and twelve rarely presented UVs (each UV was presented on 2.77% of trials). Note that the 13 participants were recruited on the basis that they were intimately familiar with only one target voice (IFV) in the presented stimuli. Thus, 12 different voices were presented in the blocks but only one voice was intimately familiar to one participant.

Participants listened to the utterance stimuli via insert earphones (*E-A-Rtone 3A*, EAR Auditory Systems) and the amplitude of the audio signal was calibrated so as to obtain peak levels of 74 dBa at the inserts. The stimuli were played back using *E-prime 1*.*0* (Psychology Software Tools). Trials were separated by an inter-stimulus interval (ISI) that varied randomly from 500 ms to 650 ms in steps of 50 ms to minimize anticipation effects. In listening to the stimuli, the participants were sitting at a distance of 180 cm from a blank computer screen with a fixation cross. They were asked to listen to the stimuli and keep their eyes on the fixation cross. For the four behavioural blocks, participants were also required to keep the fingers of their dominant hand positioned on a mouse and to indicate as quickly as possible if the voice heard during each trial was the familiar one or unfamiliar by pressing either the left or the right mouse key, respectively (this was reversed for half of the participants).

### EEG recordings and analyses

EEG signals were recorded throughout the experiment (including behavioural blocks that were not included in the EEG analyses). The recordings were performed according to the international 10–20 system and with an *ASA-lab EEG/ERP 64 channels amplifier* (ANT neuro). An online average reference was used and signals were digitized at sampling rate of 1000 Hz. Eye movements and blinks were recorded using four electrodes placed above and below the dominant eye (VEOG) and at the outer canthus of each eye (HEOG). AFz was used as ground and all other 64 channels were kept below 10 kΩ impedance during the recordings.

Offline, the recordings were band-pass filtered (0.3–30 Hz) and blinks were removed using *ASA software* (ANT neuro). All other artefacts exceeding a standard deviation of 20 μV within a sliding window of 200 ms were automatically removed with *Eeprobe GUI* (version 1.2.0.2, ANT Software). EEG recordings were then averaged across blocks and by types of voices (IFV, TV, and UV) using Fieldtrip [85], an open-source toolbox for MatLab (R2017b 9.3). Each trial in the recordings was epoched between 200 ms before and 1000 ms after each stimulus onset and the 200 ms pre-stimulus interval was used for baseline correction.

Visual inspection of the averaged signal for all conditions allowed to easily identify a P1-N1-P2 complex, directly followed by a negative deflection between 300 and 350 ms post stimulus onset, in the range of the N250, and a late positive component (LPC) extending to the end of the analysis window. Considering the data in Table 1, the P2 peak on right centro-frontal sites, the N250 peak on left fronto-central sites and the LPC on both right centro-frontal sites and left/middle centro parietal sites were of particular interest in the present study.

The P2 was peaking on frontal sites between 200 ms and 250 ms post stimuli onset. An ANOVA with three within subjects factors was carried out on the mean amplitudes between 200 and 250 ms. The factors included were voice condition (IFVs, TVs and UVs), site (F, FC, C and CP) and laterality (right and left hemispheres)

The N250 and the slow waves ERPs had a wider scope than the P2. In order to reduce statistical analyses for those two components, pools of electrodes were created to represent six scalp regions. The regions included the following electrodes and will be referred to as: middle centro-frontal (MCF; Fz, F1, F2, FCz, FC1, FC2, Cz, C1, C2), right centro-frontal (RCF; F4, F6, FC4, FC6, C4, C6), left centro-frontal (LCF; F3, F5, FC3, FC5, C3, C5), middle centro-parietal (MCP; CPz, Pz, POz, CP1, CP2, P1, P2), right centro-parietal (RCP; CP4, CP6, P4, P6, PO4, PO6) and left centro-parietal (LCP; CP3, CP5, P3, P5, PO3, PO5). Outlining electrodes were not included since ERP of interest were not elicited on those sites.

Statistical analyses were carried out using 50 ms mean amplitude samples to compare the time-course of ERP activity between experimental conditions (IFV, TV, UV) on the N250 and de slow waves ERPs. The analyses window for the N250 was between 300 ms and 350 ms while successive 50 ms mean amplitude samples were used between 450 ms and 850 ms post-stimulus onset to investigate longer slow waves ERPs. The mean amplitudes were calculated using MatLab (R2017b 9.3). Repeated measures analyses of variance (ANOVA) were then carried out using the open-source software JASP (version 0.13) with two within-subjects factors: voice condition (IFV, TV and UV) and scalp region (6 levels: MCF, RCF, LCF, MCP, RCP and LCP). Huynh-Feldt correction was applied if required and the alpha level was set at *p* < 0.05.

## Results

### Behavioural data

On the analyses of behavioural responses, all responses exceeding 1300 ms were excluded (22.30%). For the remaining trials, the overall accuracy of identification of the IFV was 98.18%. The false alarm rate, that is when either TV or UV were falsely identified as an IFV, was 0.35%. The misses, or when IFV was designated as UV, represented 1.82% of response. Most of the time, when participants made mistakes, they spontaneously informed the experimenter that they were aware of their error. These results establish that the voice stimuli were readily identified by participants.

### The P2

For the P2. the three levels repeated measures ANOVA carried out on mean amplitudes between 200 ms and 250 ms reached significance for the main effect of site, F(1.883,22.595) = 17.348, *p* < .001, η^2^ = .297 as well as for the interaction between laterality x voice conditions, F(2,24) = 3.868, *p* = .035, η^2^ = .01. Planned comparison with Bonferroni-corrected t-tests for the voice conditions on each sites revealed significant differences between IFV and TV on F4, *t*(12) = -2.3, *p* = .04 *d* = .638 and between IFV and UV on FC4, *t*(12) = 2.498, *p* = .028, *d* = .693, C4. *t*(12) = -2.25, *p* = .044, *d* = .624 and CP4, *t*(12) = -2.716, *p* = .019, *d* = .753. These results are summarized below.

### The N250

The second component was analysed using the mean amplitude between 300 ms and 350 ms post stimuli onset. In this window, the repeated measures ANOVA revealed a main effect of scalp region, F(2.78,71.397) = 12.308, *p* < .001, η^2^ = .436, and a voice condition x scalp region interaction, F(5.95,71.397) = 3.593, *p* = .004, η^2^ = .028. Planned comparison with Bonferroni-corrected t-tests for the voice conditions within each given scalp region revealed a significant difference between TV and UV in MFC, *t*(12) = -3.479, *p* = .006 and LFC, *t*(12) = -3.506, *p* = .005, *d* = -.972 regions as well as between IFV and TV in LFC, *t*(12) = 3.12, *p* = .014, *d* = .865. No other difference was observed in this time window.

Since the main difference between TVs and UVs was the varying number of presentations in the course of the experiment, two ANOVAs with within-subject factors of voice condition and region were performed, respectively on the first and second halves of the experiment, to ascertain training effects. Only the ANOVA on the second half revealed significant interaction between voice condition and region F(4.428,53.137) = 3.544, *p* = .01, η^2^ = .047. Again, Bonferroni-corrected planned comparison with voice conditions within each individual scalp region showed significant differences between TV and UV in MFC, *t*(12) = -3.215, *p* = .011, *d* = -.892 and LFC, *t*(12) = -2.624, *p* = .045, *d* = -.728 regions as well as between IFV and TV in LFC *t*(12) = 3.068, *p* = .016, *d* = .851.

### The LPC

Finally, the LPC was investigated using a 50 ms sliding window of mean amplitudes between 450 ms and 850 ms post stimuli onset. Significant voice condition x scalp region interactions were found for 500–550 ms, F(6.719,80.632) = 3.411, *p* = .003, η^2^ = .036, 600–650 ms, F(7.506,90.073) = 3.956, *p* < .001, η^2^ = .043 and 650–700 ms, F(5.98,71.754) = 2.406, *p* = .036, η^2^ = .041 time windows.

**Fig 1:**
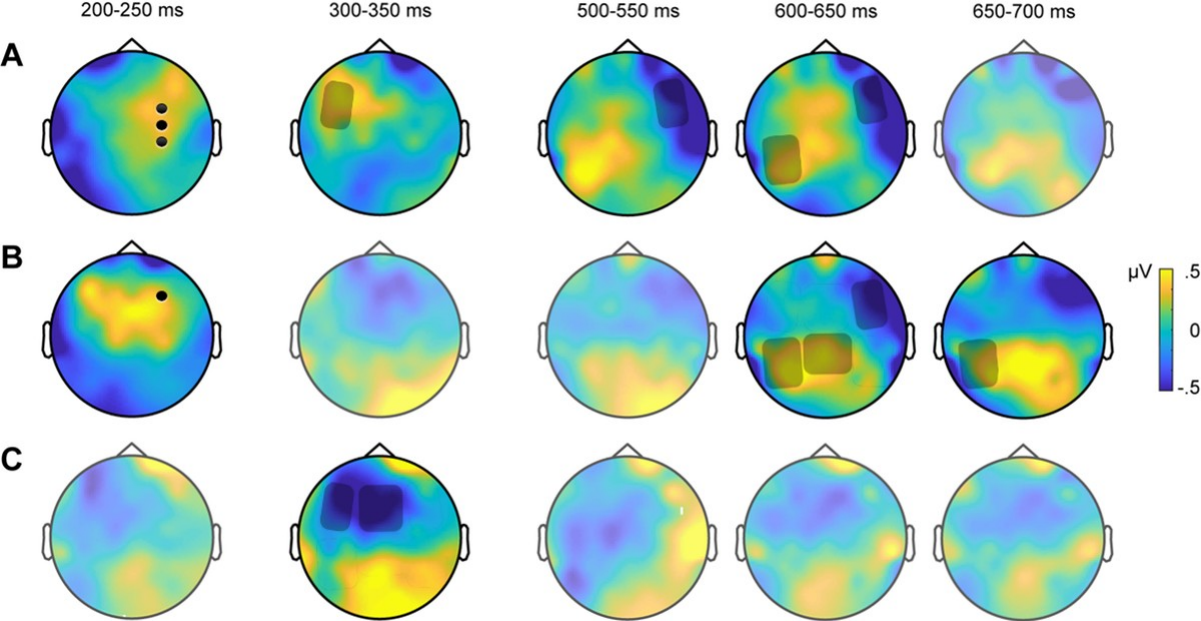
Topographic representations of the ERP differences between (A) IFVs and TVs, (B) IFVs and UVs, and (C) TVs and UVs. Darkened areas and black dots represent regions and electrodes where voice conditions were significantly different. No significant difference were found on light-shaded topographies.

As before, planned comparison were used to compare the voice conditions within each individual scalp region using the Bonferroni correction for multiple comparisons. In the 500–550 ms time window, a significant difference between IFV and TV was found in RFC, *t*(12) = -2.854, *p* = .026, *d* = -.792 and LCP, *t*(12) = -3.188, *p* = .012, *d* = .884. For the 600–650 ms time window, there was a significant difference in mean amplitudes between IFV and both TV and UV in RFC, *t*(12) = -3.056, *p* = .016, *d* = -.847, *t*(12) = -3.418, *p* = .007, *d* = -.948, and in LCP *t*(12) = 3.221, *p* = .011, *d* = .893, *t*(12) = 4.192, *p* < .001, *d* = 1.163. A significant difference between IFV and UV was also found for this time window in MPC, *t*(12) = 2.920, *p* = .022, *d* = .810. Finally, the planned comparisons for the 650–700 ms window revealed significant differences between IFV and UV in MPC, *t*(12) = 2.782, *p* = .031, *d* = .7725 and LCP, *t*(12) = 2.608, *p* = .046, *d* = .723. As one can see by observing Fig 1, IFV was significantly different from both TV and UV in the early time window (200–250 ms) and for at least two windows within the LCP. Meanwhile, both TV and UV never differed significantly with the exception of the 300–350 ms time window during which UV was also different from IFV. The main regions involved were RFC for the P2 and the LCP, the MCF and LCF for the N250 and the MCP and LCP also for the LPC.

**Fig 2:**
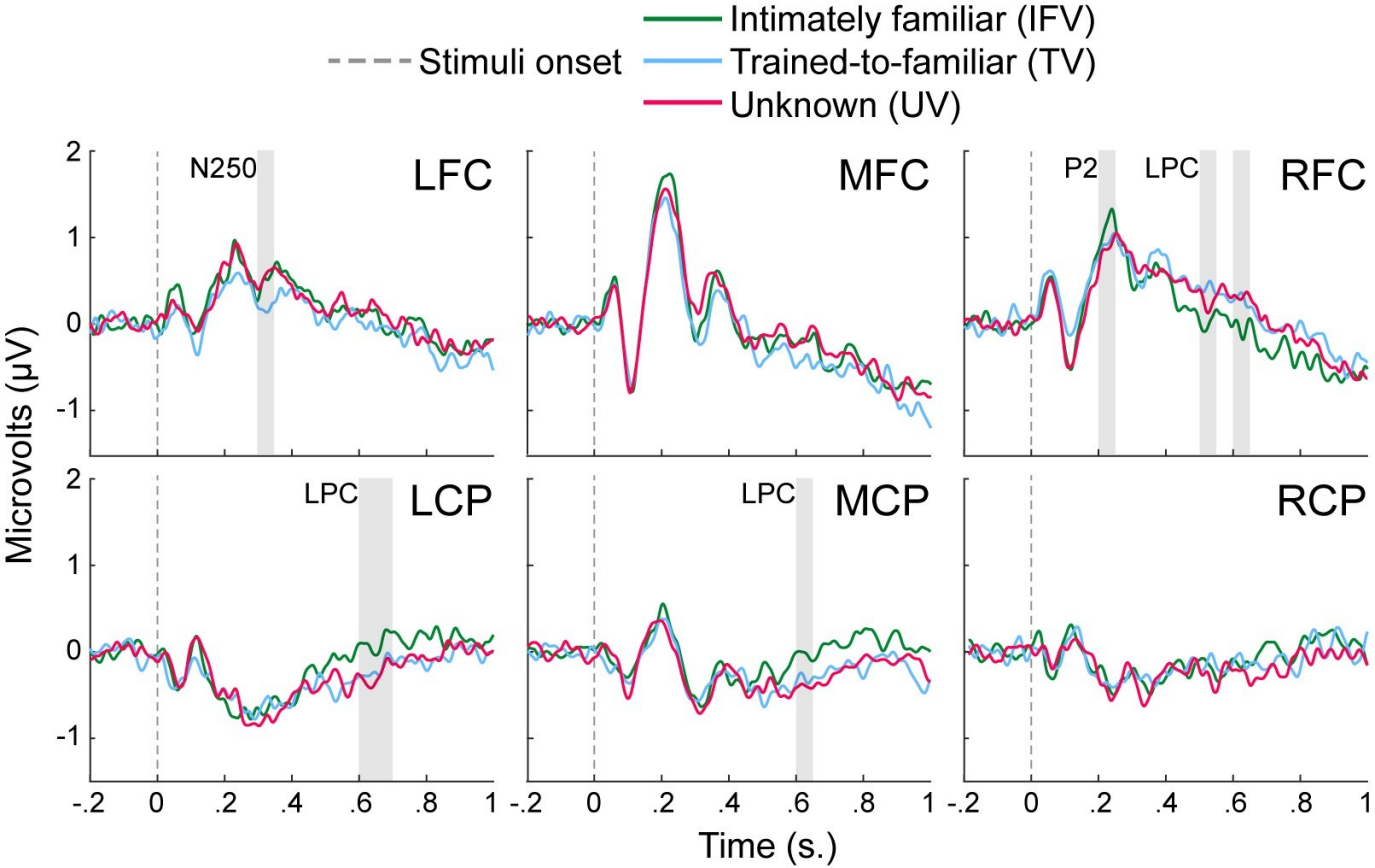
ERPs on the six regions illustrating the effects of IFVs, TVs, and UVs in the three time windows of interest. Distinct responses to IFV appear in an early window of 200–250 ms and were rightward as seen changing amplitudes at RFC, and also appeared in a late window of 500–650 ms where prolonged shifts appear in parietal sites at MPC and LCP and in frontal sites at RFC. For TVs and UVs, contrasting responses were found in a mid-late window of 300 to 350 ms, as seen in the differential responses at MFC and LFC.

To provide a broad picture of the results across the three time windows of interest, Fig 2 offers a summary of responses across the six regions. One can see that listeners’ neural responses to IFVs–where the speaker’s identity is known–stand out in the early time window in the RFC region on the P2, and in the late time window in the MPC, LCP and RFC on LPC. By contrast, listeners’ responses to TVs–for which the identity of the speaker is not known–appear in other regions, in this case at MFC and LFC regions in a mid-latency time window corresponding to the N250.

## Discussion

When one hears the voice of a close individual or a famous voice, one can “recollect” information that has to do with the identity of the speaker [31]. Intuitively, one knows *who* is speaking. This is inherently different from simply recognizing a voice as previously heard but where one may not recollect a particular speaker or “place” the voice. The purpose of this study was to substantiate this difference with respect to voice research where only some protocols distinguish between voice *recognition* and *identification* processes by reference to intimately familiar voices [e.g., 3, 56, 62, 63, 65]. In circumscribing neural responses that reflect these different processes, the use of IFVs presents an advantage in that, compared to famous voices where identity information can vary across individual listeners, there is little doubt the voice of a parent, sibling, or close-friend holds specific information on speaker identity. In this sense, the above results confirm a basic difference on processes of voice recognition and identification and suggests a time course for these processes not previously identified in the literature bearing of speaker identity processing.

Specifically, significant distinctions in ERPs were observed in three different time windows: an early response in a 200–250 ms time window associated with the P2 component, a mid-latency response at between 300–350 ms, in the N250 range, and a later occurring response between 500–700 ms. Both early- and late-latency responses were associated intimately familiar voices (in the IFV condition) compared to frequently heard or rarely heard unfamiliar voices (in the TV and UV conditions). No significant differences were observed between responses for TVs and UVs in these time windows. While some studies have also revealed such specific early ERPs and components such as MMNs for familiar voices, many have not reported later-occurring protracted responses (or LPCs) that cannot be analyzed in terms of short-time “components”. Part of the reason for this discrepancy in research findings appears to bear on the length of the stimuli. As noted previously with reference to Table 1, studies using short speech samples (single syllables) obtained results related to voice identity in a similar time range as the early responses observed in the present report. Conversely, studies in Table 1 where participants are presented with at least a few syllables have reported later-occurring responses to IFVs similar to the late responses obtained in the above results. In particular, Gonzalez, Bobes Leon [63] used stimuli lasting about 500 ms and reported responses between 280 and 840 ms post-onset. Schweinberger [65] had stimuli of 2000 ms and obtained responses ranging from 250 to 600 ms and, using stimuli of 909 ms, Schweinberger, Walther [62] reported responses from 450 to 800 ms post-onset. In the present results, short phrases averaging 793 ms elicited responses between 500 and 700 ms. There is, then, a degree of agreement in these reports on the fact that stimuli longer than a syllable associate with later-occurring responses to voices, or LPCs, that bear inherent speaker-identity information. This leads to two complementary accounts of why such responses would be drawn out beyond about 500 ms post onset.

One reason may be that accurate voice identification requires more dynamic spectro-acoustic information than what is obtained in the span of single syllables. On this possibility, the results of Plante-Hébert and Boucher [2; 2015a] showed that, although identification of intimately familiar speakers can be obtained on single syllables, quasi-perfect identification requires a few syllables. In other words, short voice samples may not provide sufficient sensory information for an associative process relating signals to a memory of speakers. In addition to this factor, a delay is likely to take place between simply recognizing sensory attributes and the associative process as the stimuli unfolds over time.

Regarding the LPCs observed in the aboce experiment, electrophysiological studies have previously shown that responses to known stimuli associated with semantic information stored in long-term memory occur later than responses to stimuli encountered before but not associated with additional contextual information [for more detail, see 86]. Moreover, the left-parietal old/new effect, associated with the recollection of semantic information about a given stimuli, is known to occur at similar latencies (500–800 ms post stimuli onset) and, as its name hints, in the left-parietal brain region. The description and latency of this left-parietal old/new effect greatly correspond to the data observed in the present experiment.

As for the mid-latency response, frequently presented voices (TV condition) elicited significantly distinct responses from rarely presented voices (UV condition). Yet this difference at mid-latencies was absent in the beginning of the experiment and grew stronger at the end. It is interesting to note that such training effects on ERPs have also been reported in studies using familiar and unfamiliar faces, as described by Tanaka, Curran [87], Herzmann, Schweinberger [88] and more recently by Wiese, Chan [89]. This evolution of responses with experience suggests specifiable neural markers of memory encoding (although heard voices in the TV condition were not accompanied by any episodic memory of particular situations involving the speakers speakers and did not include any markers of emotional expression; [90]). Thus, frequent presentations of both facial and vocal stimuli entail changing neural responses in a window of 230–320 ms post-onset. As Tanaka, Curran [87] note, there are reasons to believe that the N250 is not modality specific and can represent a developing perceptual expertise. In fact, a report by Schall, Kiebel [91] revealed that if a listener only hears a familiar voice without seeing the speaker, cortical face-processing areas are activated. The response observed also greatly corresponds, both in latency and in scalp distribution, to the well established mid-frontal old/new effect specifically associated with the feeling of having encountered a stimuli before without recalling detailed semantic information about it or knowing who is speaking [for detailled reviews, see 86, 92]. But as summarized by Young, Frühholz [93], while the recognition of faces and voices may share communalities in neural processing, auditory and visual signals have different timelines and speaker identification from heard voices alone implies a processing over a stretch of signal. We have established that, minimally, a few syllables is required for accurate identification of even intimately familiar voices [6], such that neural processing of identity information would likely reflect protracted neural components or LPCs. With this in mind, our results, combined with those of previous experiments on both speaker identity and various memory processes, suggest that given sufficient speech material, the established distinction between “remembering” or the feeling of having heard a voice before, and “knowing” who is speaking reflects in distinct neural components.

Although EEG is ill-suited to a localization of these distinct processes, the above analyses of ERP responses offer some parallels with the model of voice perception presented by Kreiman and Sidtis [39]. This model suggests a right-hemisphere processing of familiar voices as opposed to a left-hemisphere processing of unknown voices. Also, most neuroanatomical models assume that the processing of familiar voices involves the right superior temporal sulcus [e.g., 40, 94, 95]. The issue of localization is important in understanding the transition between an episodic memory of voices and the consolidation of a semantic memory of speakers, an issue that requires further research when it comes to speaker identity. However, the above results suggest that future investigations should adopt a strict distinction between voice recognition and identification in devising protocols. These terms can serve to characterize different processes and responses relating to types of vocal stimuli, such as the above categories of IFV, TV, and UV. It should also be a central consideration that the processing of identity information in voices operates on heard speech sounds that extend beyond a single syllable and that neural responses to IFVs are relatively late and drawn out. This suggests that methods which examine neural responses over stretches of speech, such as temporo-spectral coherence analyses, may be better suited to analyzing the processing of voice information than techniques that focus on short-time ERPs and their components.

## Conclusion

In short, our study offers EEG evidence supporting a distinction between processes of voice recognition and speaker identification in relation to neural markers arising at different latencies. In addition to establishing unambiguous differences between vocal recognition and identification, the preceding findings bear implications in the applied sector of forensic earwitness testimony. Traditionally, earwitness identification of speakers relies on the perceptions of listeners, which has been shown to be highly accurate, especially in the case of familiar voices [5, 6]. The present data indicate that there are, additionally, neural correlates of both familiar speaker identification and the recognition of frequently heard voices as opposed to voices that are occasionally heard. Further investigations should serve to clarify the conditions by which unfamiliar voices become highly familiar and how this relates to neural encoding processes reflecting a transition between an episodic and semantic memory of vocal information.
